# Interactions among weather and landscape affect Colorado potato beetle population dynamics

**DOI:** 10.1371/journal.pone.0345180

**Published:** 2026-03-23

**Authors:** Abigail L. Cohen, Benjamin Bradford, Russell Groves, Zsofia Szendrei

**Affiliations:** 1 Michigan State University, Department of Entomology, East Lansing, Michigan, United States of America; 2 University of Wisconsin, Department of Entomology, Madison, Wisconsin, United States of America; University of Saskatchewan College of Agriculture and Bioresources, CANADA

## Abstract

Population dynamics are controlled by life history and moderated by the environment. These factors determine the timing and magnitude of population abundance and are used to predict crop pest populations. Forecasts of pest populations typically focus on heat accumulation during the growing season when the pests are active or emerging from the soil if they have a diapause period. Weather conditions before and during diapause can impact population dynamics as well but tend to be understudied. This study used 16 years of Colorado Potato Beetle (*Leptinotarsa decimlineata*) abundance data combined with landscape and daily weather data to understand the importance of seasonal weather and landscape on predicting population abundance. Boosted-tree multiclass models predicted abundance at each life stage and across all models. Cumulative degree days, a measure of developmental time, were overwhelmingly important. To understand how the top non-temporal variables could interact with time, we used general additive mixed models to examine interactions between time and the top six ranked variables for each life stage, then generated predicted abundances across the growing season for the 10^th^, 50^th^, and 90^th^ percentiles of each variable, keeping all other variables constant. This revealed that while Colorado Potato Beetle abundance was most strongly affected by heat accumulation, other weather factors like precipitation and air saturation, as well as soil temperature during diapause, can also influence abundance trends. The only landscape variable consistently ranked in the top six was potato acreage.

## Introduction

Integrated Pest Management (IPM) relies fundamentally on understanding insect phenology, or the timing of developmental events across the growing season. Accurate predictions of when pests emerge, reproduce, and reach damaging population levels influence the success of control strategies, from insecticide applications to biocontrol agent releases [1]. Because insect development is controlled by heat accumulation and food availability, phenological models based on temperature data can forecast pest activity with remarkable precision [2]. This predictive capacity has become increasingly critical as agricultural systems face mounting challenges: widespread insecticide resistance, shifting weather patterns driven by climate change, and the need to coordinate multiple control tactics throughout extended and variable growing seasons [3]. Over the past century, the development of diverse management tools, including selective insecticides, biocontrol agents, pheromone disruption, and sterile insect techniques, have expanded our arsenal against agricultural pests, but their effective deployment hinges on phenological knowledge that reveals when insects are most vulnerable to intervention.

Colorado potato beetles (*Leptinotarsa decemlineata* Say*,* Coleoptera: Chrysomelidae) (CPB) are a prime example of this phenomenon. They are cosmopolitan pest insects and can rapidly evolve insecticide resistance [4], leading to a large body of research on their life history and how various biotic and abiotic factors influence their development [5]. However, many of these studies are lab or mesocosm-based, and we still lack a comprehensive understanding of how environmental conditions affect field populations. Colorado potato beetles overwinter in the soil as adults; their diapause is induced by a change in photoperiod and broken when soil temperatures are above 10ºC [6]. After the overwintered adults emerge from the soil, they begin feeding and laying eggs. The eggs quickly hatch, and the larvae voraciously feed, pupate in the soil, and become adults. These summer generation adults then repeat the cycle by burrowing into the soil to overwinter, typically inside or on the edge of the field where they developed. Colorado potato beetles also have facultative diapause [5] and the number of generations per season may vary, but two generations are typical [7]. Overwintering survival depends on soil temperature and fat stores, while aboveground, adult and larval survival depends on temperature, food quality and availability, and resistance to management [8]. While most adults emerge after a single winter, there are instances of prolonged dormancy with about 2% of beetles remaining in the soil for more than one winter [9].

Humidity can also influence the metabolism of overwintering insects as they restore their water balance after breaking diapause [10,11]. The magnitude of populations may vary based on the previous year’s population abundance and overwintering survival rate, as well as the ability of the beetles to disperse between fields. Because many potato growers use multi-year crop rotations, the beetle's ability to find potato resources is affected by landscape composition. While CPB have non-crop hosts, like wild *Solanum* sp., they demonstrate a strong preference for potatoes [12], but may also feed on untreated volunteer potatoes. These are potato plants that grow from leftover tubers in rotated fields and are considered a weed because they compete with rotational crops [13,14], and can act as a reservoir for CPB [15,16].

Recent research on CPB in North America has focused on insecticide resistance [17] as the most pressing question, while research on how climate and long-term weather trends affect populations is conducted primarily at the new edges of their range, mostly in Northern Europe [18–20]. While the life history of CPB in North America has been studied in the lab [21–23], and there are phenology models for the insect used in decision support [24,25], these models use lab-generated data and focus on population growth within the growing season. The only mechanism available to these models to account for differences in emergence and density caused by landscape or weather conditions outside of their active period is to alter the biofix date. Thus, we still lack an understanding of how overwintering phenology impacts population abundance during the growing season, and how landscape effects may interact with weather to influence CPB development.

To take an integrated look at how weather and landscape may affect CPB both within and across years, a 16-year dataset of weekly CPB abundance sampling across a potato-growing region in central Wisconsin, USA was combined with landscape and daily weather data. This data was then used to build a boosted-tree classification model for the adult, larval, and egg mass data, as well as the combined adult and larval data, which we called “consumers” because they both consume potato foliage. These models predicted abundance of each life stage in three classes (low, high, very high) and were then used to quantify variable importance. The highest-ranking variables for each life stage were used in generalized additive mixed models (GAMMs). Finally, the models were fed simulated data to generate hypothetical predictions of abundance using the 10^th^, 50^th^, and 90^th^ percentile values of each variable, keeping all other variables constant, to examine how changes in weather and landscape affect field populations of CPB over time. We hypothesized that variables related to soil conditions in the periods where CPB are entering and maintaining diapause are important, as they affect overwintering mortality and emergence. We also hypothesized that air moisture conditions may influence CPB abundance by affecting egg and larval mortality. We expected potato cover and composition to be important, based on previous research, but hypothesized that there may be other land cover types that are associated with CPB if they are more commonly grown the year before potato, or are more hospitable to volunteers. The method and timing of tillage can affect volunteer emergence, and the approved herbicides that can effectively manage volunteers vary by crop [26].

## Methods and materials

### 2.1. Sampling data

**Fig 1:**
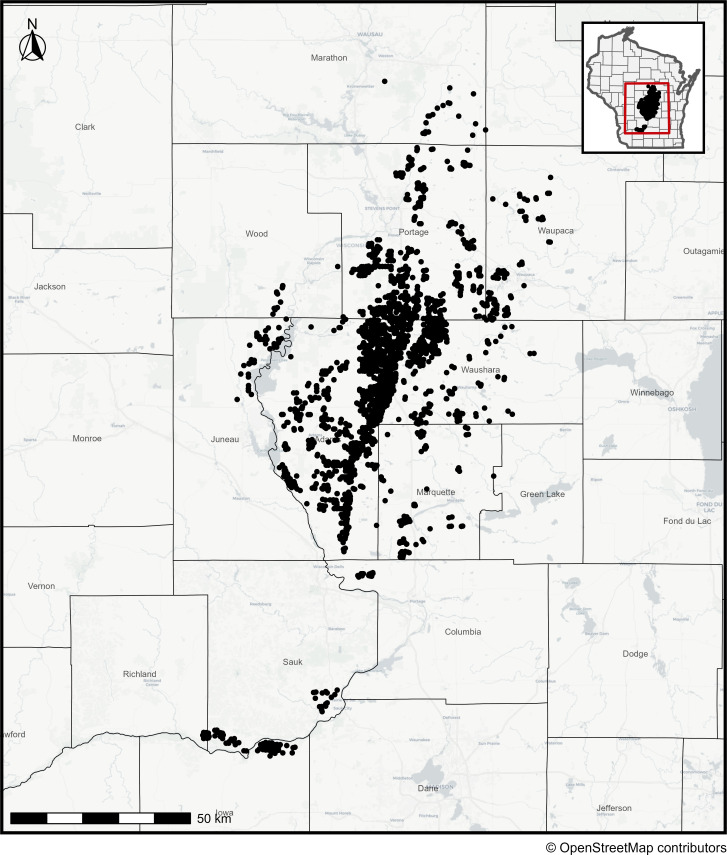
Map of Wisconsin, USA, showing Colorado potato beetle sampling locations over time. Egg masses, larvae, and adult stages were sampled between 2008 and 2023 in commercial potato fields.

The CPB abundance data used to construct the models come from a Wisconsin-based pest scouting company, Pest Pros Crop Consultants (a division of Allied Cooperative), from 2004 to 2023. There were 200–400 fields sampled per year, except in 2012–2013, which had 9–12 fields due to lost data during the paper to digital record-keeping transition at the company (S1 Table). All fields sampled were managed using conventional management practices. Scouting was performed weekly in commercial potato fields in the Central Sands region, which is the main potato growing area (~12,000 km^2^) in Wisconsin, USA (Fig 1), and extends from a latitude of 43.15° to 44.81° N and a longitude of −90.2° to −89.00° E. Across all years, the mean Euclidian nearest neighbor (ENN) distance is 338 ± 139 m (mean ± SE), as fields in this region are typically spaced on 400 m centers or smaller. Adults and larvae were collected by field scouts with sweep nets and reported as averages per 25 sweeps, with scouts sweeping 6–12 interior sites and 4–12 exterior sites per field, depending on field size. Pupae were not sampled, as that life stage takes place belowground in the soil. Egg masses were assessed independently of sweeps; at each within-field sampling site, five plants were randomly chosen and visually inspected for egg masses and reported as the number of egg masses per plant.

### 2.2. Landscape and climate data

Landscape (crop composition) data were extracted from the USDA NASS Cropland Data Layer (CDL) [27]. This data was available from 2008 to present; data from 2008-2023 was used in the analysis. The CDL raster was reclassified using the Food and Agriculture Organization (FAO) crop classification guide [28], keeping potato, corn, soybean, and wheat, as crop classes. These crops were singled out because they are the crops most commonly rotated with potatoes in the study region [29], and therefore most likely to harbor overwintering CPB and volunteer potatoes. Non-soybean crops (dry beans and peas) were binned together as ‘Legume’ (they were separated from soybean because soybean is a much more common crop), and the remaining cereal crops were binned as ‘Other Cereals’. Alfalfa and hay crops were binned together as ‘Forage’. All other crops were binned into the ‘Other Crop’ class. All bodies of water were combined into a ‘Water’ class, all developed land was combined into a ‘Developed’ class, and all non-crop, non-developed land was combined into a ‘Semi-Natural’ class. For each cover class, two landscape metrics were considered, and measured in a 1 km circular radius around each sampling point using the R package ‘landscapemetrics’, [30]: total area in hectares and perimeter-area (P:A) ratio. The Euclidean nearest-neighbor distance to potato and the Shannon diversity index of each site was also calculated using the eleven reclassified crop and non-crop categories.

The air temperature, precipitation, and snow water data were downloaded using the daymetr package [31], which draws daily weather data from the DAYMET database [32], which has a spatial resolution of 1 km. Degree days were calculated with a biofix date of January 1^st^ using the single sine method and horizontal cutoff. The lower temperature threshold was 11 °C and the upper threshold was 31 °C [22,24]. The minimum and maximum daily vapor pressure deficit (VPD) data were sourced from PRISM daily data [33] with a spatial resolution of 800m. Vapor pressure deficit is a measure of the difference between the amount of water the air can hold and how much it actually holds; a low VPD indicates saturated air and high humidity and a high VPD indicates dry air and low humidity. The daily mean soil temperature data were sourced from the Copernicus Climate Change Service ERA5 post-processed daily statistics [34], which has a spatial resolution of 0.25° (~ 19.67 km at 45° latitude). We used the soil temperature level 2, which represents the soil at 7–28 cm, because CPB typically burrow to 10–25 cm in the soil during diapause [5].

To estimate the timing of CPB emergence and generation periods, we used a generalized linear model (GLM)/ generalized additive model (GAM) approach [35,36]. First, a GLM with a Poisson distribution was fitted, which included some fixed effects to control for known sources of variation as well as Cumulative Degree Days (CDD) as a random effect term. From this model, CDD coefficients were extracted and used to fit a GAM, which generated a smooth curve through the coefficients. The point at which the smooth curve first crossed from negative to positive was considered the start of colonization, the maxima of each hump was the peak emergence of that generation, the minima was the breakpoint between generations, and the end of the season was when the curve crossed back from a positive to a negative value (S1 Fig). While Colorado potato beetle generations may briefly overlap in the field, the minima identify the point at which generational turnover occurs. For each year of data (January 1^st^-December 31^st^), winter/spring was defined as the period before emergence (<203 CDD), the first generation period was from emergence to the first minima (203–936 CDD), the second generation period was from the first minima to the second minima (936–1800 CDD), and the fall/winter period was everything after the second minima until CDD stopped accumulating on December 31^st^ (>1800 CDD). While there was a 3^rd^ generation identified at 1971 CDD (S1 Fig), we did not include a third generation period in the analysis because it did not occur consistently across years (S2 Fig).

Almost all the sampling data fell within the first- and second-generation periods, with small amounts of count data occurring in the fall/winter diapause period (S2 Fig). However, since the adults that emerge in a given spring entered the soil in the previous calendar year, we use the fall/winter data from the previous year. Thus, for the first year of sampling data, 2008, the fall/winter weather associated with it was from 2007. The variables considered per period were median daily minimum (Tmin) and maximum temperature (Tmax) (°C), median daily soil temperature (°C), median daily minimum (VPDmin) and maximum (VPDmax) vapor pressure deficit (hPa), mean total precipitation (mm), mean solar radiation (W/m^2^), and mean snow water equivalent (kg/m^2^) from 2007-2023.

### 2.3. Data analysis

A machine learning model is used to analyze the relative importance of the weather and landscape variables because it is uniquely suited to analyzing large numbers of explanatory variables [37,38]. Similar analyses used a classification approach to model insect data [38,39], so this analysis used a multiclass model of abundance with three levels of abundance class: “Low”, “High”, and “Very High”. For adults, larvae, and consumers, the Low class was < 25^th^ percentile of abundance per site/day, the High class was between the 25^th^ and 75^th^ percentile, and the Very High class was > 75^th^ percentile. The egg data was zero-inflated (S3 Fig), likely due to the challenges in scouting, so the egg data were split using the 10^th^ and 90^th^ percentiles (S2 Table).

The data was checked for cross-correlation, and variables with a correlation > 0.7 were removed (S4-S6 Fig). Each life stage was split 75/25 into training and testing data. The tidymodels package [40] was used to prepare the data and run the models. In the preparation stage, each set of life stage data had explanatory variables normalized for ease of comparison. The classification models used an extreme-boosted gradient tree engine (xgboost) [41], and all hyperparameters were tuned using a Latin hypercube design with 30 combinations and ten folds. The best fitting model was determined by area under the receiver operating characteristic curve (AUC), and the accuracy and Brier class of the best fit model were assessed (S2 Table). Accuracy is the proportion of the data that is correctly predicted, while AUC measures the ratio of true to false positives and is more indicative of predictive quality. Brier class scores are a measurement of error and assess the mean squared difference between the predicted probability of an outcome and the actual outcome. All metrics are on a 0–1 scale.

**Fig 2:**
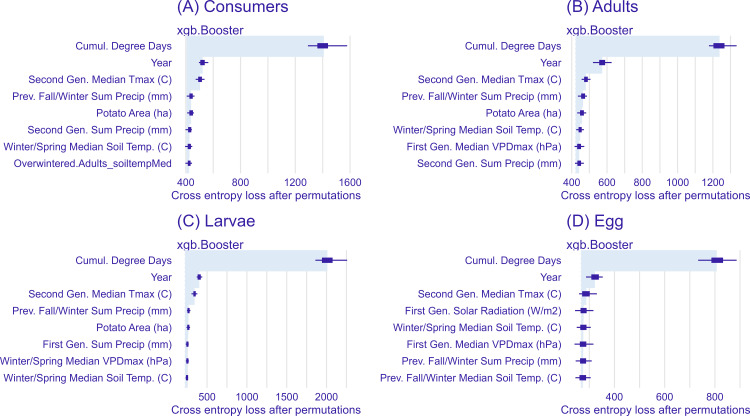
Variable importance for the tree-based multiclass models. The eight most important temporal, spatial, and weather factors influencing Colorado potato beetle are shown for (A) consumer (adults and larvae), (B) adult, (C) larva, and (D) egg mass abundance. Importance is indicated by the change in model performance when each variable is dropped from the model. All variables were normalized for ease of comparison.

The variable importance of each model was assessed using the DALEXtra R package [42], which measures the relative contribution of each variable to the model outcome by dropping variables from the full model and assessing the loss in model performance. The loss function used for multiclass models is cross-entropy loss, and results were averaged over 50 permutations. The higher the value, the more important the variable is to the model (Fig 2). The top six non-temporal variables were then used to construct GAMMs. As the tree-based models showed that the temporal variables of CDD and year were by far the most important, a GAMM approach allowed for an assessment of how the weather and landscape affected each life stage over time.

To separate the effect of each variable from its interaction with CDD, each non-temporal variable was represented as both a linear parametric term and as a smoothed interaction with CDD, with data normalized using the scale function in the base R package [43]. Cumulative degree day was a separate smooth term, to measure its effect separately, and year was a parametric term, to quantify abundance trends across years. Sampling location was treated as a factor and was represented in the models as a random effect to account for potential management differences between sites. Each life stage was modeled with a Poisson distribution family, which is commonly used when working with count data. Within GAMMs, a significant parametric term indicates that the variable influences life stage abundance across the entire season, and a significant smooth term indicates that there is an effect of time. Some variables have both a significant parametric and smooth term, indicating that the variable has both an overall effect and a CDD-mediated effect on life stage abundance, while a non-significant parametric term and a significant smooth term indicate that the variable may only be influencing abundance at certain times in the growing season. To test how each interaction affects the abundance of each life stage, the GAMMs were used to generate predictions for each life stage for any given site, keeping all variables but the selected one constant over the entire sampling period. For each focal variable, predictions were generated for the 10^th^, 50^th^, and 90^th^ percentiles of its range and averaged across all years of sampling, then plotted over CDD.

## Results

### 3.1. Gradient-boosted modelling

The life stage models performed similarly, with AUCs in the range of 0.864-0.871 (S2 Table, S7 Fig). The accuracy ranged from 0.649-0.862, and the egg mass model had the highest accuracy while the adult model had the lowest accuracy. Brier class values ranged from 0.098-0.208, and the egg mass model had the lowest value while the adult model had the highest value (S2 Table). The models were best at predicting the Low and Very High class, and worst at predicting the High class (S7 Fig), although there was less difference among classes in the egg model, which may explain why this model had the best accuracy and Brier scores, but the lowest AUC. The egg mass data had a right-skewed distribution and lower abundance compared to adults and larvae (S3 Fig).

When ranking variables, CDD has the highest relative importance in all models, and year is the second-highest ranked variable in all models (Fig 2). Across the models, 21 of the 24 variables were weather-related, and 3 of the 24 were landscape-related, with second-generation period median Tmax and potato area ranking the highest among the consumer, larval, and adult models. Within the 21 weather-related variables, 5 were from the first-generation period, 5 were from the second-generation period, 5 were from the winter/spring diapause period, and 6 were from the previous fall/winter (Fig 2).

### 3.2. Generalized additive mixed models

**Fig 3:**
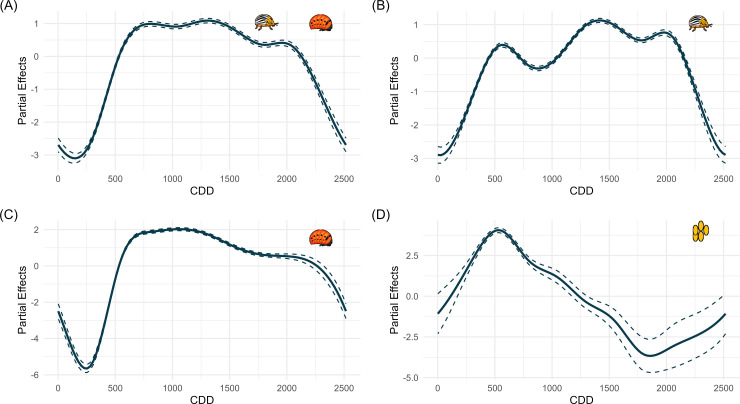
Partial effects plots on the link scale for the Cumulative Degree Day (CDD) smooth term in each life stage model. The life stages are (A) consumer, (B) adult, (C) larva, and (D) egg masses. The line represents the effect of the CDD smooth term on the model alone, with all other terms set to their mean. The dotted lines are the 95% confidence interval (CI). Each model uses a Poisson distribution with a log-link scale.

The GAMMs had R^2^ values that ranged from 17.3-40.9% and had 55.1-58.3% deviance explained. There was a significant positive effect of the linear year term in the consumer, adult, and larva models, and a significant negative effect of year on the egg model (S3-S6 Tables). To understand abundance trends over time, the partial effects of the CDD smooth term on abundance of each life stage were modeled. The linear predictor values increase after approximately 250 CDD for consumers (Fig 3A), slightly later than the start of colonization generated by the GLM/GAM approach. The first-generation adult peak was around 500 CDD (Fig 3B), which corresponds with increasing larval and egg abundance (Figs 3C,3D) and is slightly earlier than the first-generation peak generated by the GLM/GAM approach. There is a second peak at ~1400 and a third, less distinct, peak at 2000 CDD and then a sharp decline (Fig 3B). This suggests that the third generation is less distinct than the first two and may be inconsistent, which is also seen in the GLM/GAM approach (S1 Fig). The larval linear predictor values began to increase as soon as diapause ended at ~250 CDD and started to slowly taper off at 1000 CDD. After adults emerge from diapause, they still need to mate and lay eggs, and it takes approximately 120 CDD for eggs to develop into first instar larvae [24], so there should be a gap between the emergence of adults and the emergence of larvae. This overlap between adults and larvae is most likely a product of natural variation in adult emergence across the sampling period and variation in when sampling began across years (S1 Table). This also explains the lack of two distinct larval generations: the first larval generation is extended by late-emerging adults and overlaps with the partial second larval generation (Fig 3C). The egg mass model had a distinct peak around 500 CDD and declines until 1750 CDD, where it begins to increase again, but the confidence intervals are much wider for this period. (Fig 3D).

In the consumer model, it is only the previous fall/winter precipitation and soil temperature variables that did not have a significant parametric term; all other terms were significant (S3 Table). In the adult model, the previous fall/winter precipitation terms, as well as the winter/spring and first-generation period VPDmax parametric terms, were not significant (S4 Table). In the larval model, only previous fall/winter precipitation parametric term was not significant (S5 Table). In the egg model, the first-generation solar radiation and second-generation Tmax parametric terms were significant (S6 Table). Across all models, all smooth terms were significant, but instead of an estimated effect size, they were evaluated using the estimated degrees of freedom (EDF). The closer an EDF is to zero, the less it affects model performance, with an EDF = 0 meaning the variable has no effect [44]. Our models used a reference EDF of 8 for the CDD smooth term and 9 for the CDD interaction terms, and the lowest EDF across all models was 5.903, with most EDFs in the 7–8 range.

#### 3.2.1. Consumer model.

**Fig 4:**
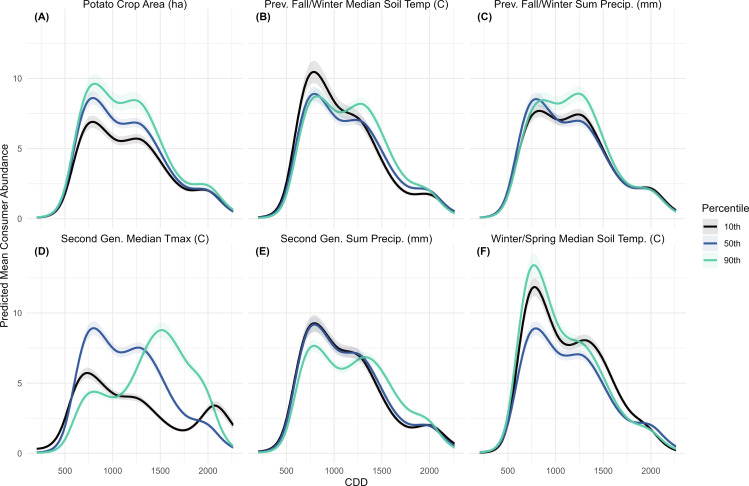
Colorado potato beetle consumer models with predicted mean consumer abundance over Cumulative Degree Days (CDD). Each panel (A-F) represents predicted abundance at the response scale across the sampling period for each title variable at the 10th, 50th and 90th percentile, when all other variables are held constant at their mean. Predicted abundance was calculated for all sampling years (2008-2023) then averaged. The shaded areas represent a 95% confidence interval.

The consumer model predicted a two-peaked abundance curve, with the relative size of the peaks varying depending on the manipulated variable (Fig 4). When second-generation period Tmax was varied, a distinct second peak occurred under the 90^th^ percentile scenario at 1500 CDD, while the 10^th^ percentile scenario showed a smaller, later second peak near 2000 CDD. The 50^th^ percentile (mean) scenario showed a larger first peak and a smaller second peak at 1250 CDD, followed by a sharp decline (Fig 4D). These results confirmed that heat accumulation was the primary factor influencing the turnover between CPB generations, because in prediction scenarios where other variables are manipulated, abundance magnitude changes but not the overall pattern of development. When potato area (Fig 4A) was varied, abundance was highest under the 90^th^ percentile scenario, although the timing and relative size of the generational peaks did not differ across scenarios. Manipulating soil temperature produced intervals where the 10^th^ percentile scenario was associated with higher abundance than the warmer scenarios, though this pattern was limited to the first peak. For previous fall and winter soil temperature, the 50^th^ and 90^th^ percentile scenarios overlapped during the first peak, but after 1250 CDD, the 90^th^ percentile consistently predicted the highest abundance (Fig 4B). When manipulating winter and spring soil temperature, the 90^th^ percentile scenario produced the highest abundance during the first peak, the 10^th^ percentile scenario was somewhat lower, and the 50^th^ percentile scenario was significantly lower. Around 1250 CDD, abundance under the 10^th^ percentile scenario slightly exceeded the others, after which the 50^th^ and 90^th^ predicted abundances converged (Fig 4F). The smallest differences among scenarios occurred when precipitation variables were varied. For previous fall and winter precipitation predicted abundances overlapped during the first peak, with slightly higher abundance under the 90^th^ percentile scenario after 1000 CDD (Fig 4C). For second-generation period precipitation, there was little difference between the 10^th^ and 50^th^ percentile scenarios, both of which had higher predicted abundance than the 90^th^ percentile until 1250 CDD, when the 90^th^ percentile scenario became the most abundant (Fig 4E).

#### 3.2.2. Adult model.

**Fig 5:**
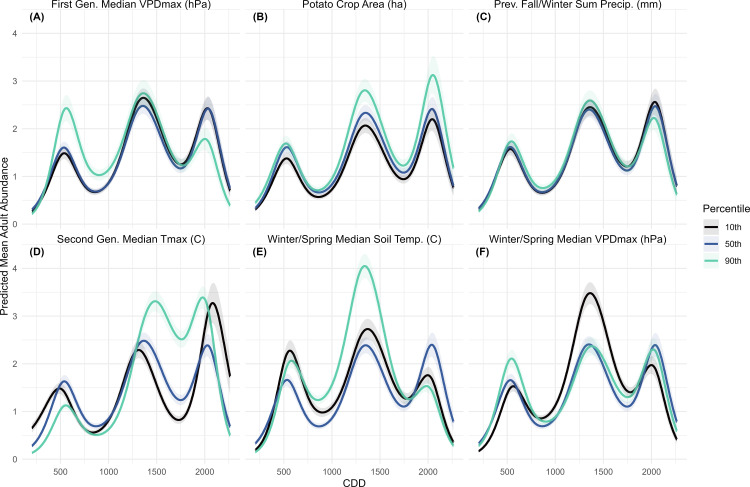
Colorado potato beetle adult models with predicted mean adult abundance over Cumulative Degree Days (CDD). Each panel (A-F) represents predicted abundance at the response scale across the sampling period for each title variable at the 10th, 50th and 90th percentile, when all other variables are held constant at their mean. Predicted abundance was calculated for all sampling years (2008-2023) then averaged. The shaded areas represent a 95% confidence interval.

The adult model predicted a small peak of first-generation adults, followed by larger second- and third-generation peaks (Fig 5). Varying the second-generation period Tmax affected both the timing and size of the second and third generation peaks. Under the 90^th^ percentile scenario, the second peak occurred slightly later, and adult abundances declined moderately before the third peak. In contrast, under the 50^th^ and 10^th^ percentile scenarios, abundance dropped sharply between the second and third peaks at around 2000 CDD, with the most distinct third peak observed under the 10^th^ percentile scenario (Fig 5D). Potato-growing area also influenced predicted abundance (Fig 5B), especially during the second generation, although there was no difference between the 50^th^ and 10^th^ percentile scenarios. When first-generation period VPDmax was varied, the 90^th^ percentile scenario (representing the driest air) produced the highest first-generation peak, but this effect did not persist in later generations, and by the third peak, the 90^th^ percentile scenario had the lowest abundance (Fig 5a). Predicted abundance also varied with winter and spring soil temperature across scenarios. The 10^th^ and 90^th^ percentile scenarios produced the highest first-generation peaks, the 90^th^ had the highest second-generation peak, and the 50^th^ percentile had the highest third-generation peak (Fig 5E). An opposite trend occurred when winter and spring VPDmax was varied: the 90^th^ percentile scenario produced the highest in the first peak, the 10^th^ percentile the highest second-generation peak, and all scenarios showed similar abundance in the third generation (Fig 5F). Manipulating the previous fall and winter precipitation values did not affect abundance across scenarios (Fig 5C).

#### 3.2.3. Larval model.

**Fig 6:**
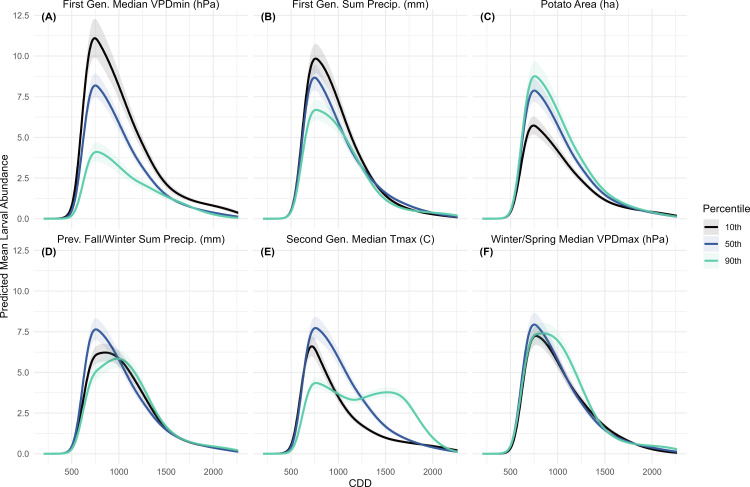
Colorado potato beetle larvae models with predicted mean larval abundance over Cumulative Degree Days (CDD). Each panel (A-F) represents predicted abundance at the response scale across the sampling period for each title variable at the 10th, 50th and 90th percentile, when all other variables are held constant at their mean. Predicted abundance was calculated for all sampling years (2008-2023) then averaged. The shaded areas represent a 95% confidence interval.

The predicted larval abundance curve featured a single main peak between 500 and 1500 CDD with a gradual decline extending through the remainder of the season (Fig 6). As a result, there was no clear distinction between larvae that developed into the second-generation adults and those that contributed to the third generation. When second-generation Tmax was varied, predicted larval abundance was lower under both the 10^th^ and 90^th^ percentile scenarios and highest under the median scenario. However, under all scenarios, larval abundance persisted later into the season and did not decline until ~ 1750 CDD (Fig 6E). This may explain why the consumer model predicted the highest abundance at the 50^th^ percentile in the first peak but then aligned more closely with the adult model in the second peak (Fig 4E). When first-generation period VPDmin was manipulated (Fig 6A), predicted abundance showed an inverse relationship with percentile. The opposite trend occurred when first-generation precipitation was varied (Fig 6B), with the highest larval abundance under the lowest rainfall scenario. Beyond the first-generation period, moisture conditions were less influential, as differences in abundance and peak timing between minimal across scenarios for previous fall and winter precipitation (Fig 6D) and winter and spring VPDmax (Fig 6F). The effect of potato area mirrored that observed in the adult and consumer models, influencing overall abundance similarly (Fig 6C).

#### 3.2.4. Egg model.

**Fig 7:**
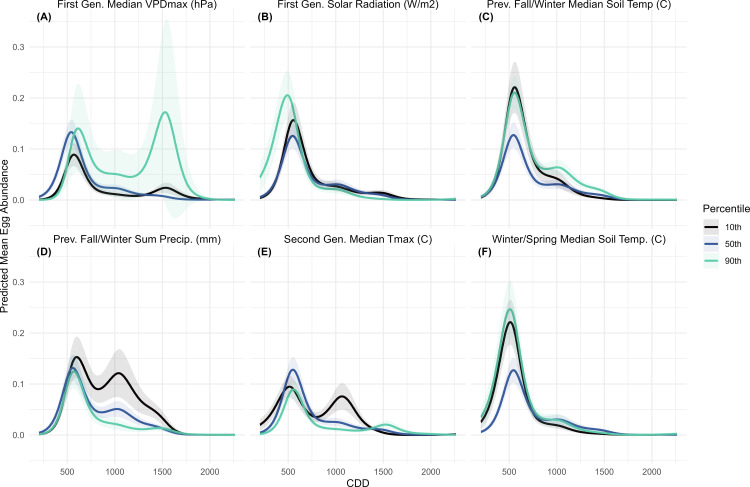
Colorado potato beetle egg mass models with predicted mean egg mass abundance over Cumulative Degree Days (CDD). Each panel (A-F) represents predicted abundance at the response scale across the sampling period for each title variable at the 10th, 50th and 90th percentile, when all other variables are held constant at their mean. Predicted abundance was calculated for all sampling years (2008-2023) then averaged. The shaded areas represent a 95% confidence interval.

This model had a lower R^2^ than those for the other life stages (S6 Table) and showed greater variability in predicted abundance. There are also no scenarios where egg abundance increases after 1750 CDD, as we see in the partial effects plots (Fig 3D). This variance likely resulted from the structure of the dataset, which contained more zero values and less variation among non-zero responses (S2 Fig). The most distinct response was observed for first-generation VPDmax, which produced a second peak at ~1500 CDD under the 90^th^ percentile scenario, although the 95^th^ confidence interval was large (Fig 7A). Other scenarios also produced a second egg peak, but these were smaller, occurred earlier, and had narrower confidence intervals. A second peak was also predicted in the 10^th^ percentile scenario for second-generation Tmax (Fig 7E) and under the 10^th^ percentile of previous fall and winter precipitation (Fig 7D). In both cases, the peaks occurred earlier, around 1000 CDD, and their confidence intervals did not overlap with those of the other scenarios. When winter and spring (Fig 7F) or previous fall and winter soil temperature (Fig 7C) were varied, predicted abundances were similar for both the 10^th^ and 90^th^ percentile scenarios, while the mean scenario showed lower abundance. Manipulating first-generation solar radiation produced a higher and slightly earlier first egg peak under the 90^th^ percentile scenario (Fig 7B).

## Discussion

These results show that while CPB population development in commercial potato fields is mostly driven by heat accumulation post-diapause, landscape context and pre-diapause climate conditions also influenced the magnitude and rate of population growth. Among the broad range of landscape and seasonal climate variables tested, only a few had clear effects on predicted abundance, and only manipulation of second-generation period Tmax led to notable differences in the timing of population development. This suggests that predictive models of CPB risk could incorporate surrounding potato area and winter temperatures to improve estimates of expected CPB pressure on crops. However, once emergence begins, heat accumulation remains the most dominant factor in determining the buildup of field populations. There appears to be a strong influence of air saturation on larval abundance, which is important for accessing CPB risk because larvae are the most voracious feeders [21] and coincide with the stage when potato plants are smaller and more vulnerable. In contrast, first-generation adults emerging from the soil had the highest predicted abundance under the least saturated winter/spring VPDmax scenario (90^th^ percentile). This trend did not carry into the second generation, where higher predicted adult abundance occurred under the most saturated air scenario (10^th^ percentile; Fig 5F). Because this pattern was not linked to carryover from the first generation, or increased larval abundance (Fig 6F), the underlying mechanism remains unclear.

Winter/spring soil temperature also influenced predicted abundance between the first and second generations, suggesting that diapause conditions may affect post-emergence development. Soil moisture and humidity are known to affect CPB emergence from the soil [45,46], but few studies have examined how environmental conditions experienced during diapause affect post-emergence populations. For example, an experiment found that manipulating soil temperatures during diapause of bean leaf beetles produced inconsistent effects on their overwintering survival, though warmer winters led to earlier emergence [47]. Other factors outside of landscape and weather may also affect CPB emergence and development: systemic neonicotinoid exposure caused a difference in fatty acid composition which led to a delay in emergence compared to susceptible CPB [48].

This analysis aimed to clarify how overwintering phenology influences CPB abundance. Although eleven diapause-period variables ranked highly in the importance analysis, only manipulation of winter/spring median soil temperature produced strong differences in predicted consumer (Fig 4F) and adult (Fig 5F) abundances. While previous fall/winter precipitation ranked as an important variable in the consumer, adult, and egg models (Fig 2), it only produced a distinct effect on egg abundance – and the effect was small overall (Fig 7D). The effects of previous fall/winter soil temperature were subtler, but the highest consumer abundance during the first peak occurred under the coldest scenario and was followed by a steeper decline after 1000 CDD (Fig 4B). Previous research studies have linked overwintering survival to soil temperature [49] and soil insulation [50], though those studies only measured adult survival, rather than subsequent population trends. The apparent positive effect of cold fall/winter soil temperatures on CPB overwintering may be because a colder median temperature at the end of a growing season induces diapausing beetles to burrow deeper into the soil, decreasing overwintering mortality [51], while a colder soil during diapause delays diapause termination and reduces exposure to early spring cold snaps. There may also be indirect effects of cold winter temperatures, as other competing herbivorous insects and natural enemies may also have higher overwintering mortality. However, beetles also experience a refractory phase in which they do not immediately respond to temperature increases after diapause termination [6]. These findings suggest that diapause conditions influence the magnitude of the first adult generation but do not persist in the second generation, which is more strongly affected by summer Tmax (Fig 4D). This potential relationship between colder soil temperatures and increased diapause depth may also explain the effects of winter and spring soil temperature on consumers, where both the low and high scenarios produced higher abundance in the first peak compared to the mean temperature (Fig 4F).

The predicted egg mass and larva abundances were mediated by both moisture and temperature, though there are no strong differences in predicted egg abundance. There was a slight increase in predicted egg abundance under high solar radiation (Fig 7B), and colder second-generation temperatures produced a second egg generation (Fig 7E). This may reflect delayed development of the first adult generation, resulting in a later or more extended egg-laying period. When precipitation was manipulated, predicted egg abundance was highest under the low-rainfall scenario (Fig 7D). Similarly, larval abundance was highest under the low-rainfall scenario in the first-generation, but in contrast, it was also highest under the 10^th^ percentile VPDmin (most saturated air) scenario during the same period. This suggests that larvae benefit from humid air but may suffer during rainy conditions. When humidity is experimentally manipulated, low humidity was found to increase larval mortality, particularly in the early instars [52]. Thus, the difference in predicted abundance when first-generation VPDmin was manipulated may stem from reduced mortality among smaller, more desiccation-prone larvae, though the study region rarely experiences relative humidity as low as the experimental conditions in that study. High precipitation can also reduce the efficiency of certain neonicotinoid seed treatments [53], and may interfere with larval feeding if larvae are dislodged from plants.

While we hypothesized that crops commonly rotated with potatoes might harbor volunteer potatoes or act as a source of CPB, only potato area emerged as a top-ranked model variable (Fig 2). This aligns with previous research demonstrating the importance of potato intensity in driving CPB population dynamics. Rotation distance from the nearest previous potato field can affect CPB adult abundance and colonization risk [54], and contributes to the development of neonicotinoid resistance [55]. While potato area did not affect egg mass abundance, it had a clear positive relationship with both adult and larval abundance. Possibly, greater surrounding potato acreage increases connectivity and makes immigration of adults between fields easier, decoupling adult density from local egg density. Non-crop land cover variables, like semi-natural area, also did not rank highly in importance, though other research has found that semi-natural habitat can negatively affect CPB abundance. One study found that proximity to native prairie grasses was associated with higher predation on CPB [56], and a previous study in our sampling region found a negative correlation between abundance and surrounding grassland cover [54]. The hypothesized mechanism for this effect is that beetles in potato fields surrounded by grassland could not successfully disperse into more protected overwintering habitats and suffered higher overwintering mortality.

These results suggest that warmer temperatures, especially in the summer and winter, could increase overwintering survival, extend second generation activity, and promote the formation of a partial third generation. However, they also suggest that colder fall/winter temperatures could lead to higher overwinter survival, which suggests that as climate change leads to more extreme weather, Colorado potato beetles will thrive. Moderate climate warming scenarios also demonstrate accelerated CPB development and allow for an additional generation [57]. Warmer winters were associated with higher predicted first-generation adult abundance (Fig 5F), and warmer summers produced greater and longer-lasting second-generation abundance (Fig 5D). Although winter/spring soil temperature were not ranked as important variables for larvae, larval abundance was higher under the 50^th^ and 10^th^ percentile second-generation Tmax scenarios than the 90^th^. However, the 90^th^ percentile scenario extended larval persistence in the field (Fig 6e). The higher abundance under lower Tmax scenarios and the relationship between VPDmin and predicted abundance (Fig 6A) suggest that cooler, more humid conditions support larval development by reducing desiccation stress. Heat wave experiments similarly showed that extreme heat reduced larval survival, but survivors developed faster and reached larger sizes [58]. The 90^th^ percentile of second-generation period Tmax remains below the species’ upper developmental threshold of 31 °C (Table 1), indicating that further warming could still enhance CPB population growth before heat stress becomes limiting.

**Table 1:** The variables used in each life stage GAMM model and the values that correspond to their 10^th^, 50^th^, and 90^th^ percentiles.

Life stage	Variable	Percentile		
		10th	50th	90th
**Consumer**	Second Gen. Median Tmax (°C)	24.74	26.6	28.49
	Prev. Fall/Winter Sum Precip. (mm)	188.94	285.99	560.23
	Potato Area (ha)	11.21	50.35	109.99
	Winter/Spring Median Soil Temp. (°C)	−1.001	−0.27	0.72
	Second Gen. Sum Precip. (mm)	106.67	180.82	313.23
	First Gen. Median VPDmin (hPa)	0.04	0.82	1.26
**Adult**	Second Gen. Median Tmax (°C)	24.74	26.6	28.49
	Prev. Fall/Winter Sum Precip. (mm)	188.94	285.99	560.23
	Potato Area (ha)	11.21	50.35	109.99
	Winter/Spring Median Soil Temp. (°C)	−1.001	−0.27	0.72
	First Gen. Median VPDmax	16.21	18.72	24.54
	Winter/Spring Median VPDmax	2.69	3.59	4.50
**Larvae**	Second Gen. Median Tmax (°C)	24.74	26.6	28.49
	Prev. Fall/Winter Sum Precip. (mm)	188.94	285.99	560.23
	Potato Area (ha)	11.21	50.35	109.99
	Winter/Spring Median VPDmax	2.69	3.59	4.50
	First Gen. Median VPDmin (hPa)	0.04	0.82	1.26
	First Gen. Sum Precip. (mm)	120.73	209.90	284.75
**Egg**	Second Gen. Median Tmax (°C)	24.74	26.65	28.48
	First Gen. Solar Radiation (W/m^2^)	362.09	376.57	390.26
	Winter/Spring Median Soil Temp. (°C)	−1.001	−0.27	0.72
	Prev. Fall/Winter Sum Precip. (mm)	188.94	285.99	560.23
	First Gen. Median VPDmax (hPa)	16.21	18.72	24.54
	Prev. Fall/Winter Median Soil Temp (°C)	2.67	6.60	10.44

Variable values shown here are not normalized.

## Conclusion

Cumulative degree days were expected to be key predictors in the machine learning models, serving as a measure of accumulated heat over time. The results confirm that a degree-day-based model effectively predicts abundance classes, supporting the validity of existing forecasting approaches for CPB. Our models also suggest that a third generation of CPB adults can occur, and an avenue of future research is to better understand what conditions are required for a third generation, and common they are in the field. However, purely prediction-focused models may not fully capture the interactions among temporal, weather, and landscape factors. This analysis evaluated a broad suite of weather and landscape variables to identify additional factors that could improve predictions of CPB population trends and phenology. Future versions of CPB forecasting models may benefit from incorporating soil temperature and moisture to better estimate overwintering survival and emergence, and air moisture to predict larval mortality. Moisture conditions may also indirectly affect mortality by mediating the activity of the soil biome, including entomopathogenic fungi [59] and nematodes [60]. Warming conditions could lead to higher and more sustained CPB abundance, increasing pest pressure on potato production. Warmer winters may also intensify challenges from weeds [61] and pathogens [62], potentially compounding management demands on growers. Understanding how CPB responds to temperature and humidity extremes is also relevant to predicting how other temperate pests may adapt under climate change.

This analysis used both air and soil temperature, though most current predictive models focus solely on air temperature. As soil temperature modeling improves and errors decrease at greater depths [63], it will become easier to integrate soil microclimate data into phenology models and track insect development across all life stages. Integrating microclimatic variables like humidity and soil temperature into CPB forecasts could enable more precise timing of control measures and reduce reliance on prophylactic insecticide applications like at-plant noenicitinoid treatments. Because most CPB diapause research has been conducted in laboratory or experimental settings, linking soil temperature data to field populations would significantly expand our understanding of CPB life history and improve the accuracy of predictive models.

## Supporting information

S1 Fig A model of Colorado potato beetle adult phenology across cumulative degree days. Points represent log-scaled abundance data used to build the model, and the smoothed curve shows the predicted abundance over time. The point where the curve first crosses from negative to positive indicates the start of colonization, each peak represents the emergence maximum for a generation, the minima is the breakpoint between generations, and the end of the season occurs where the curve returns from positive to negative values.
(TIF)

S2 Fig Histograms of raw count data from all years analyzed, divided by the seasonal periods used in the analysis. Data to the right of the dashed line fall within the corresponding period. Winter/Spring Diapause corresponds to 0–203 CDD, the Overwintered Gen. to 203–936 CDD, Summer Generation to 936–1800 CDD, and Fall/Winter Diapause to values exceeding 1800 CDD.
(TIFF)

S3 Fig Histograms of abundance for each life stage. (TIFF)

S4 Fig Pearson correlation plot between all the weather variables considered in the machine learning models of Colorado Potato Beetle life stage abundance. Variables were selectively removed so that the maximum correlation was 70%.
(TIFF)

S5 Fig Pearson correlation plot between the final set of weather variables considered in the machine learning models of Colorado Potato Beetle life stage abundance. This shows the set of weather variables used for analysis, with highly correlated (> 0.7) variables were removed.
(TIFF)

S6 Fig Pearson correlation plot between all the landscape variables considered in the machine learning models of Colorado Potato Beetle life stage abundance. No landscape variables met the correlation threshold for removal.
(TIFF)

S7 Fig Receiver operating characteristic (ROC) curves for the multiclass tree-based models. Each panel shows results for the (A) consumer, (B) adult, (C) larval, and (D) egg mass models. For consumers, adults, and larvae, the “low” class represents values below the 25th percentile of abundance per site/day, the “high” class spans the 25th–75th percentiles, and the “very high” class exceeds the 75th percentile. For egg masses, the “low” class is below the 10th percentile, the “high” class spans the 10th–90th percentiles, and the “very high” class is above the 90th percentile.
(TIFF)

S1 Table A summary table of sampling effort. For each year of sampling, it shows the total number of sites sampled, total mean abundance for each life stage, and the start and end sampling dates/day of year.
(DOCX)

S2 Table The hyperparameters of the best-fit machine learning model for Colorado potato beetle life stages. Model iterations are determined by the number of trees. The minimum child weight controls node partitioning: higher values make the model more conservative (default 1). The tree depth controls model complexity, with higher values increasing the risk of overfitting (default = 6). The learn rate controls step size shrinkage and influences overfitting; higher values increase this risk (default = 0.3). Loss reduction governs model partitioning, with higher values producing a more conservative model (default = 0). Model performance was evaluated using accuracy (range of 0−1), Area Under the receiver-operating characteristic Curve (AUC) (0−1), and Brier class (1−0).
(DOCX)

S3 Table Model summary for the Colorado potato beetle consumer generalized additive mixed model (GAMM). Each model term is defined using the type, shrinkage, and H_null_ interpretation columns. Parametric terms are assessed using the estimate of the coefficient and standard error (SE). Smooth terms are assessed using the estimated degrees of freedom (EDF) and the reference degrees of freedom (ref. DF). Furthermore, the smooth terms have a concurvity value, which measures the degree to which a smooth term could be approximated by other smooth terms in the model. Statistically significant terms are bold.
(DOCX)

S4 Table Model summary for the adult generalized additive mixed model (GAMM). Each model term is defined using the type, shrinkage, and H_null_ interpretation columns. Parametric terms are assessed using the estimate of the coefficient and standard error (SE). Smooth terms are assessed using the estimated degrees of freedom (EDF) and the reference degrees of freedom (ref. DF). Furthermore, the smooth terms have a concurvity value, which measures the degree to which a smooth term could be approximated by other smooth terms in the model. Statistically significant terms are bold.
(DOCX)

S5 Table Model summary for the larval generalized additive mixed model (GAMM). Each model term is defined using the type, shrinkage, and H_null_ interpretation columns. Parametric terms are assessed using the estimate of the coefficient and standard error (SE). Smooth terms are assessed using the estimated degrees of freedom (EDF) and the reference degrees of freedom (ref. DF). Furthermore, the smooth terms have a concurvity value, which measures the degree to which a smooth term could be approximated by other smooth terms in the model. Statistically significant terms are bold.
(DOCX)

S6 Table Model summary for the egg mass generalized additive mixed model (GAMM). Each model term is defined using the type, shrinkage, and H_null_ interpretation columns. Parametric terms are assessed using the estimate of the coefficient and standard error (SE). Smooth terms are assessed using the estimated degrees of freedom (EDF) and the reference degrees of freedom (ref. DF). Furthermore, the smooth terms have a concurvity value, which measures the degree to which a smooth term could be approximated by other smooth terms in the model. Statistically significant terms are bold.
(DOCX)
