# Supplementary figures and images for: Interactions among weather and landscape affect Colorado potato beetle population dynamics
Source: PLoS One. 2026 Mar 23;21(3):e0345180. doi: 10.1371/journal.pone.0345180 (PMC13008058; doi:10.1371/journal.pone.0345180)

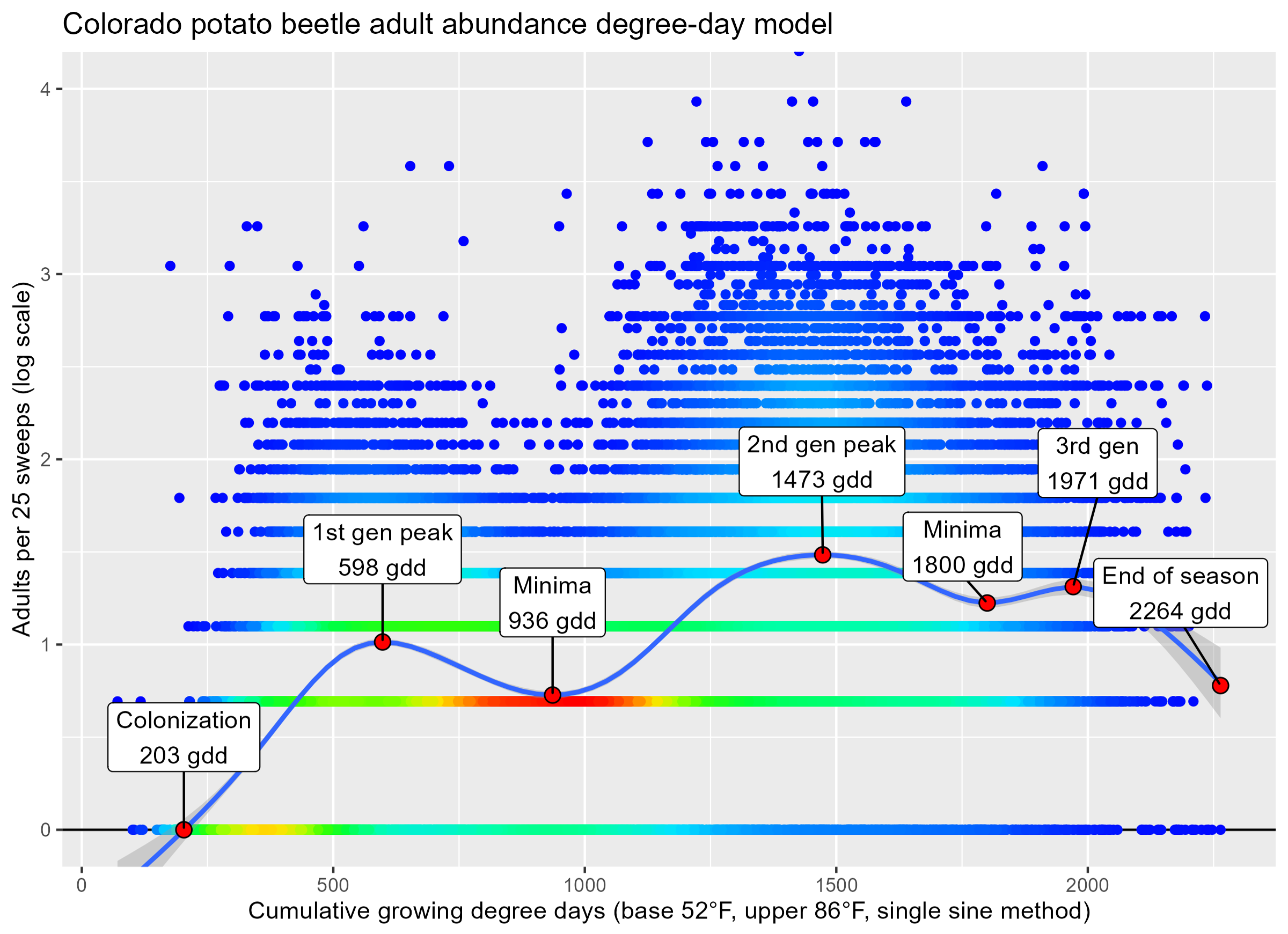

Supplement: S1 Fig — Points represent log-scaled abundance data used to build the model, and the smoothed curve shows the predicted abundance over time. The point where the curve first crosses from negative to positive indicates the start of colonization, each peak represents the emergence maximum for a generation, the minima is the breakpoint between generations, and the end of the season occurs where the curve returns from positive to negative values. (TIF) [file pone.0345180.s001.tif]

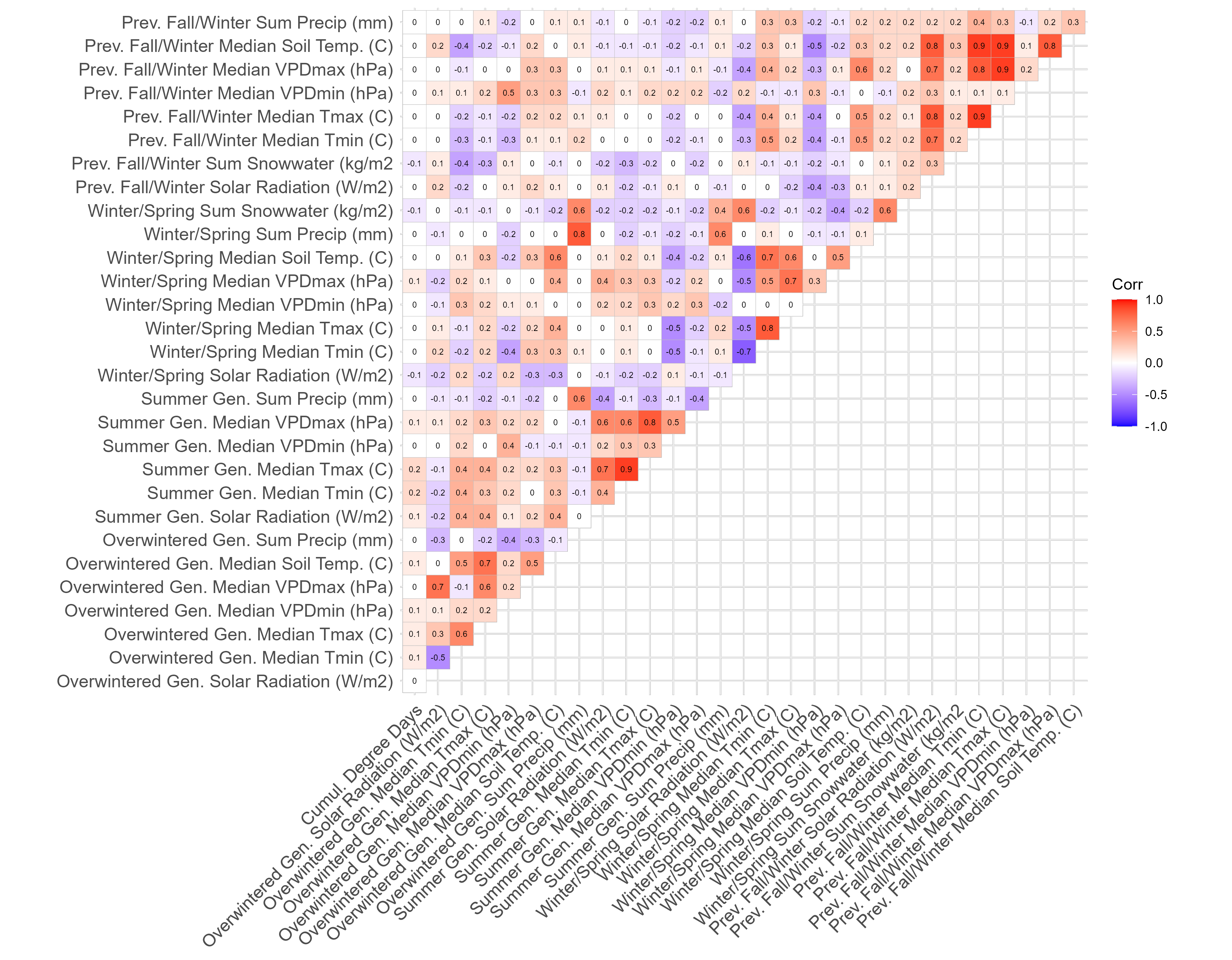

Supplement: S4 Fig — Variables were selectively removed so that the maximum correlation was 70%. (TIFF) [file pone.0345180.s004.tiff]

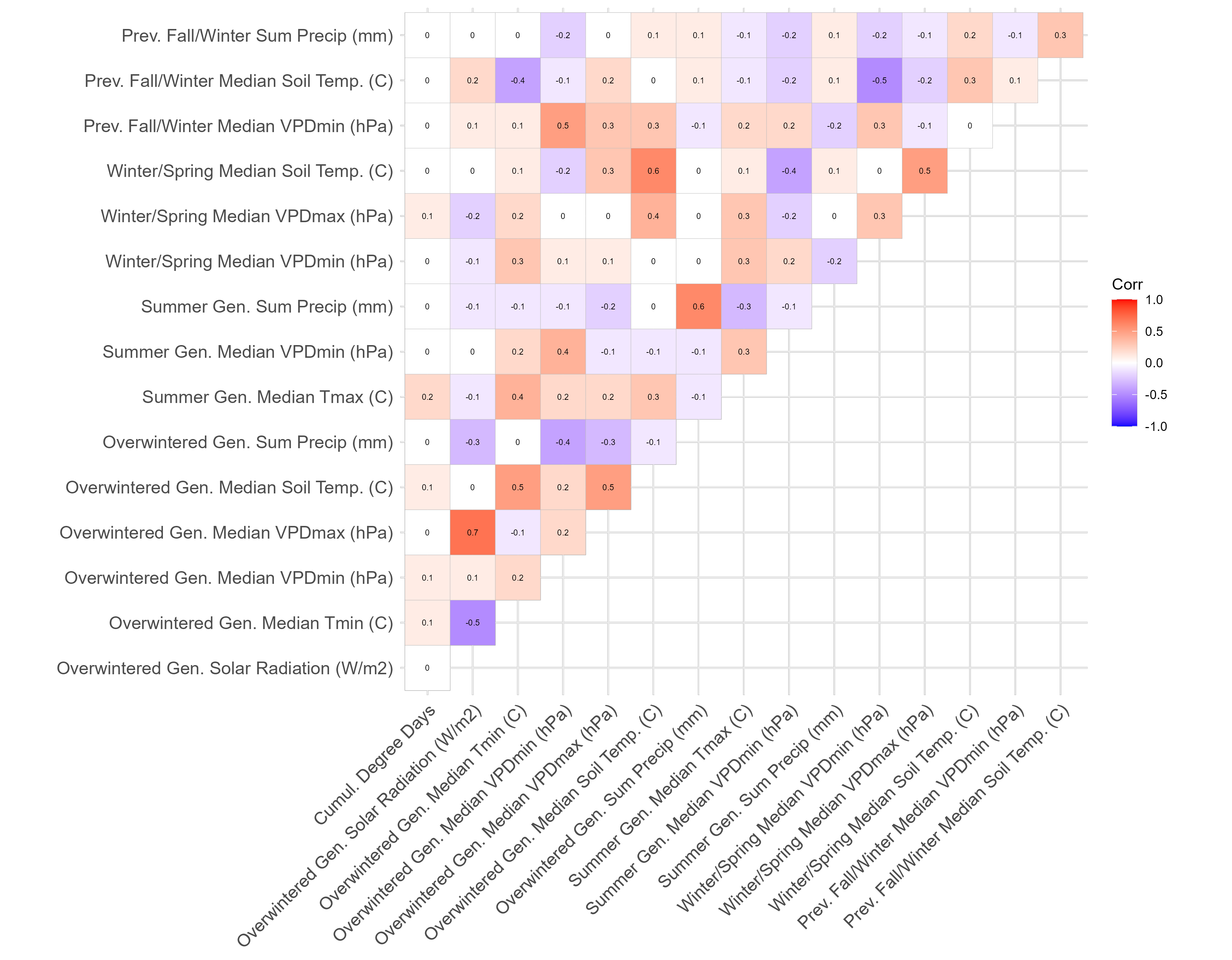

Supplement: S5 Fig — This shows the set of weather variables used for analysis, with highly correlated (> 0.7) variables were removed. (TIFF) [file pone.0345180.s005.tiff]

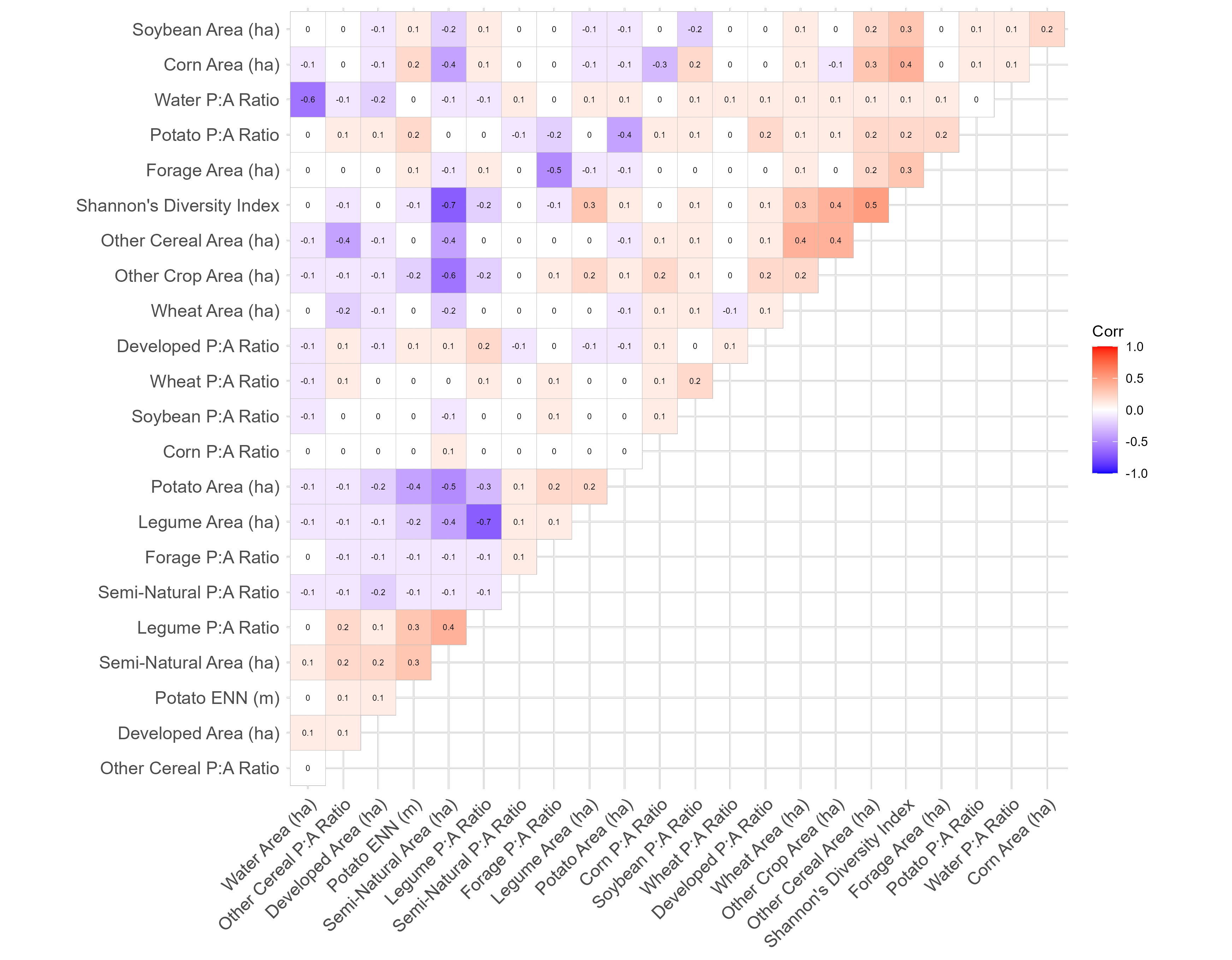

Supplement: S6 Fig — No landscape variables met the correlation threshold for removal. (TIFF) [file pone.0345180.s006.tiff]

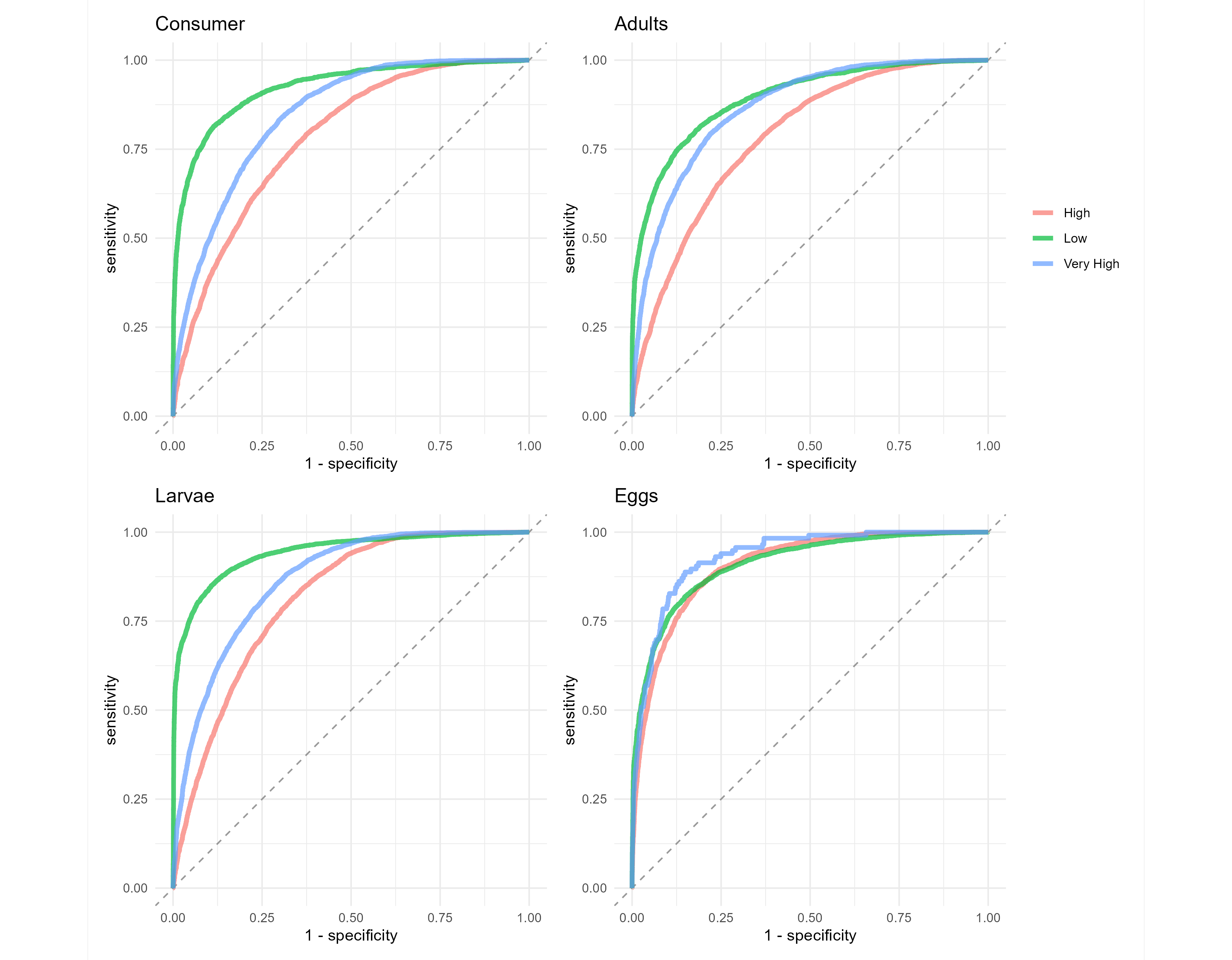

Supplement: S7 Fig — Each panel shows results for the (A) consumer, (B) adult, (C) larval, and (D) egg mass models. For consumers, adults, and larvae, the “low” class represents values below the 25th percentile of abundance per site/day, the “high” class spans the 25th–75th percentiles, and the “very high” class exceeds the 75th percentile. For egg masses, the “low” class is below the 10th percentile, the “high” class spans the 10th–90th percentiles, and the “very high” class is above the 90th percentile. (TIFF) [file pone.0345180.s007.tiff]
