# Supplementary material for: Interactions among weather and landscape affect Colorado potato beetle population dynamics
Source: PLoS One. 2026 Mar 23;21(3):e0345180. doi: 10.1371/journal.pone.0345180 (PMC13008058; doi:10.1371/journal.pone.0345180)
Supplement: S1 Table — For each year of sampling, it shows the total number of sites sampled, total mean abundance for each life stage, and the start and end sampling dates/day of year. (DOCX) [file pone.0345180.s008.docx]

| **Year** | **# Sites** | **Total Mean Abundance** | | | **Sampling Dates** | | **Sampling Day of Year** | |
| --- | --- | --- | --- | --- | --- | --- | --- | --- |
|  |  | *Adults* | *Larva* | *Eggs* | *First* | *Last* | *First* | *Last* |
| 2008 | 235 | 4799.7 | 6402 | 641.3 | 3-Jun | 28-Aug | 155 | 241 |
| 2009 | 284 | 4156 | 6401.6 | 983.6 | 3-Jun | 27-Aug | 154 | 239 |
| 2010 | 225 | 3171.4 | 6613 | 281 | 2-Jun | 1-Sep | 153 | 244 |
| 2011 | 228 | 3996.4 | 7828.7 | 348.8 | 8-Jun | 10-Sep | 159 | 253 |
| 2012 | 12 | 302.6 | 792.8 | 34.3 | 20-Apr | 3-Aug | 111 | 216 |
| 2013 | 9 | 506 | 1005.7 | 5.4 | 1-Jun | 15-Aug | 152 | 227 |
| 2014 | 285 | 6356.4 | 12232 | 135.2 | 7-Aug | 9-Sep | 219 | 252 |
| 2015 | 280 | 8273.6 | 17008.7 | 187.8 | 8-May | 25-Aug | 128 | 237 |
| 2016 | 301 | 15139.1 | 26578.2 | 260.3 | 24-May | 14-Aug | 145 | 227 |
| 2017 | 333 | 13888.4 | 26304.7 | 319 | 17-Aug | 29-Aug | 229 | 241 |
| 2018 | 422 | 15841.7 | 27488.4 | 281.6 | 18-May | 5-Sep | 138 | 248 |
| 2019 | 374 | 11861.7 | 14697.2 | 102.6 | 10-Jun | 17-Jul | 161 | 198 |
| 2020 | 340 | 13097.2 | 16422.5 | 189.28 | 9-Jun | 17-Feb | 161 | 204 |
| 2021 | 335 | 10494.4 | 14749 | 152.8 | 14-Jun | 18-Aug | 165 | 230 |
| 2022 | 328 | 11732.2 | 16343.4 | 174.62 | 14-Jun | 29-Aug | 165 | 241 |
| 2023 | 338 | 11500.2 | 13976.1 | 193.8 | 13-Jun | 9-Aug | 164 | 221 |
