# Supplementary material for: Interactions among weather and landscape affect Colorado potato beetle population dynamics
Source: PLoS One. 2026 Mar 23;21(3):e0345180. doi: 10.1371/journal.pone.0345180 (PMC13008058; doi:10.1371/journal.pone.0345180)
Supplement: S2 Table — Model iterations are determined by the number of trees. The minimum child weight controls node partitioning: higher values make the model more conservative (default 1). The tree depth controls model complexity, with higher values increasing the risk of overfitting (default = 6). The learn rate controls step size shrinkage and influences overfitting; higher values increase this risk (default = 0.3). Loss reduction governs model partitioning, with higher values producing a more conservative model (default = 0). Model performance was evaluated using accuracy (range of 0−1), Area Under the receiver-operating characteristic Curve (AUC) (0−1), and Brier class (1−0). (DOCX) [file pone.0345180.s009.docx]

Table S2.

| **Life Stage** | **Class Breakdown** | **Trees** | **Minimum child weight** | **Tree**  **depth** | **Learning**  **rate** | **Loss**  **reduction** | **Accuracy** | **AUC** | **Brier Class** |
| --- | --- | --- | --- | --- | --- | --- | --- | --- | --- |
| Consumers | Low: ≤ 0.25  High: (0.25, 9)  Very High: > 9 | 585 | 25 | 10 | 0.0399 | 1.97E-9 | 0.697 | 0.846 | 0.203 |
| Adults | Low: ≤ 0.2  High: (0.2, 2.9)  Very High: > 2.9 | 585 | 25 | 10 | 0.0399 | 1.97E-9 | 0.649 | 0.858 | 0.208 |
| Larvae | Low: 0  High: (0, 5)  Very High: > 5 | 1641 | 8 | 15 | 0.00765 | 7.24E-4 | 0.716 | 0.871 | 0.194 |
| Eggs | Low: 0  High: (0, 1)  Very High: > 1 | 1386 | 26 | 6 | 0.0111 | 0.0498 | 0.862 | 0.864 | 0.098 |
