# Supplementary material for: Interactions among weather and landscape affect Colorado potato beetle population dynamics
Source: PLoS One. 2026 Mar 23;21(3):e0345180. doi: 10.1371/journal.pone.0345180 (PMC13008058; doi:10.1371/journal.pone.0345180)
Supplement: S3 Table — Each model term is defined using the type, shrinkage, and Hnull interpretation columns. Parametric terms are assessed using the estimate of the coefficient and standard error (SE). Smooth terms are assessed using the estimated degrees of freedom (EDF) and the reference degrees of freedom (ref. DF). Furthermore, the smooth terms have a concurvity value, which measures the degree to which a smooth term could be approximated by other smooth terms in the model. Statistically significant terms are bold. (DOCX) [file pone.0345180.s010.docx]

Table S3.

| Term | Type | Shrinkage | H_null_ interpretation | Estimate | SE | EDF | ref. DF | | | p | Concurvity | |  |
| --- | --- | --- | --- | --- | --- | --- | --- | --- | --- | --- | --- | --- | --- |
| **Intercept** | **Parametric** | **No** | **No effect** | **-83.820** | **5.900** |  | |  | **<0.001** | | |  | |
| **Second Gen. Median Tmax (C)** | **Parametric** | **No** | **No effect** | **-0.079** | **0.015** |  | |  | **0.007** | | |  | |
| **Potato Area (ha)** | **Parametric** | **No** | **No effect** | **0.088** | **0.013** |  | |  | **<0.001** | | |  | |
| **Winter/Spring Median Soil Temp. (C)** | **Parametric** | **No** | **No effect** | **0.079** | **0.015** |  | |  | **<0.001** | | |  | |
| Prev. Fall/Winter Sum Precip. (mm) | Parametric | No | No effect | -0.010 | 0.013 |  | |  | 0.470 | | |  | |
| **Second Gen. Sum Precip. (mm)** | **Parametric** | **No** | **No effect** | **-0.83** | **0.014** |  | |  | **<0.001** | | |  | |
| Prev. Fall/Winter Median Soil Temp. (C) | Parametric | No | No effect | 0.030 | 0.016 |  | |  | 0.0654 | | |  | |
| **Year** | **Parametric** | **No** | **No effect** | **0.042** | **0.003** |  | |  | **<0.001** | | |  | |
| **Cumulative Degree Days (CDD)** | **Smooth** | **Yes** | **No effect** |  |  | **7.986** | | **8** | **<0.001** | | | **0.520** | |
| **Second Gen. Median Tmax × CDD** | **Smooth** | **Yes** | **No effect** |  |  | **8.862** | | **9** | **<0.001** | | | **0.476** | |
| **Potato Area × CDD** | **Smooth** | **Yes** | **No interaction** |  |  | **8.371** | | **9** | **0.017** | | | **0.303** | |
| **Winter/Spring Median Soil Temp. × CDD** | **Smooth** | **Yes** | **No interaction** |  |  | **8.790** | | **9** | **<0.001** | | | **0.452** | |
| **Prev. Fall/Winter Sum Precip. × CDD** | **Smooth** | **Yes** | **No interaction** |  |  | **8.768** | | **9** | **<0.001** | | | **0.742** | |
| **Second Gen. Sum Precip. × CDD** | **Smooth** | **Yes** | **No interaction** |  |  | **8.752** | | **9** | **<0.001** | | | **0.695** | |
| **Prev. Fall/Winter Median Soil Temp. × CDD** | **Smooth** | **Yes** | **No interaction** |  |  | **8.757** | | **9** | **<0.001** | | | **0.488** | |
| **Site** | **Random** | **Yes** | **No random effect** |  |  | **4078.537** | | **4323** | **<0.001** | | | **0.004** | |
| **R^2^** | 40.8% |  |  |  |  |  | |  |  | | |  | |
| **Deviance explained** | 57.1% |  |  |  |  |  | |  |  | | |  | |
| **N** | 52309 |  |  |  |  |  | |  |  | | |  | |
